# Discontinuous Patterns of Brain Activation in the Psychotherapy Process of Obsessive-Compulsive Disorder: Converging Results from Repeated fMRI and Daily Self-Reports

**DOI:** 10.1371/journal.pone.0071863

**Published:** 2013-08-15

**Authors:** Günter Schiepek, Igor Tominschek, Stephan Heinzel, Martin Aigner, Markus Dold, Annemarie Unger, Gerhard Lenz, Christian Windischberger, Ewald Moser, Martin Plöderl, Jürgen Lutz, Thomas Meindl, Michael Zaudig, Oliver Pogarell, Susanne Karch

**Affiliations:** 1 Institute of Synergetics and Psychotherapy Research, Paracelsus Medical University, Christian Doppler University Hospital, Salzburg, Austria; 2 Clinic of Psychosomatic Medicine, Windach/Ammersee and Munich, Germany; 3 Psychiatric University Hospital St. Hedwig, Charité Campus Mitte, Berlin, Germany; 4 Department of Psychiatry and Psychotherapy, Medical University of Vienna, Austria; 5 MR Centre of Excellence, Center for Medical Physics and Biomedical Engineering, MedicalUniversity of Vienna, Austria; 6 Department of Suicide Prevention, Paracelsus Medical University, Christian Doppler University Hospital, Salzburg, Austria; 7 Institute of Clinical Radiology, Ludwig-Maximilians-University Munich, Germany; 8 Department of Psychiatry and Psychotherapy, Ludwig-Maximilians-University Munich, Germany; Bellvitge Biomedical Research Institute-IDIBELL, Spain

## Abstract

This study investigates neuronal activation patterns during the psychotherapeutic process, assuming that change dynamics undergo critical instabilities and discontinuous transitions. An internet-based system was used to collect daily self-assessments during inpatient therapies. A dynamic complexity measure was applied to the resulting time series. Critical phases of the change process were indicated by the maxima of the varying complexity. Repeated functional magnetic resonance imaging (fMRI) measurements were conducted over the course of the therapy. The study was realized with 9 patients suffering from obsessive-compulsive disorder (subtype: washing/contamination fear) and 9 matched healthy controls. For symptom-provocative stimulation individualized pictures from patients’ personal environments were used. The neuronal responses to these disease-specific pictures were compared to the responses during standardized disgust-provoking and neutral pictures. Considerably larger neuronal changes in therapy-relevant brain areas (cingulate cortex/supplementary motor cortex, bilateral dorsolateral prefrontal cortex, bilateral insula, bilateral parietal cortex, cuneus) were observed during critical phases (order transitions), as compared to non-critical phases, and also compared to healthy controls. The data indicate that non-stationary changes play a crucial role in the psychotherapeutic process supporting self-organization and complexity models of therapeutic change.

## Introduction

With a lifetime prevalence of 2–3% [1], [2] and a median prevalence for the total population of also 2–3% [3], obsessive-compulsive disorder (OCD) is one of the most frequent adult psychiatric disorders, often showing a chronic or recurrent course [4]. The impact of obsessions and compulsions on a person’s quality of life is considerable. In addition to the symptoms themselves, which are subdivided into different subtypes such as washing/contamination fear, controlling/checking, symmetry/ordering, hoarding, and aggressive, sexual, or religious thoughts (obsessions) [5], [6], a number of neuropsychological impairments have been described (e.g., concerning attention, task and contingency shifts, suppression of intrusive thoughts and irrelevant information, complexity management, visuospatial information processing, working memory, action and conflict monitoring as well as implicit learning) [7], [8].

Neurobiological models assume an impaired serotonin and dopamine metabolism [9] especially in the fronto-striato-thalamic system [10], [11]. Within these fronto-striato-thalamic loops, different feedback mechanisms are interacting with each other [12], [13]. The indirect loop allows for projection inhibition from thalamic to cortical regions and thus for situational appropriate and flexible behavior. It appears that in OCD patients these inhibitions of thalamo-cortical projections originating at the striatum (putamen, nucleus caudatus, nucleus accumbens) are shifted in favor of the direct and activating loop.

**Figure 1:**
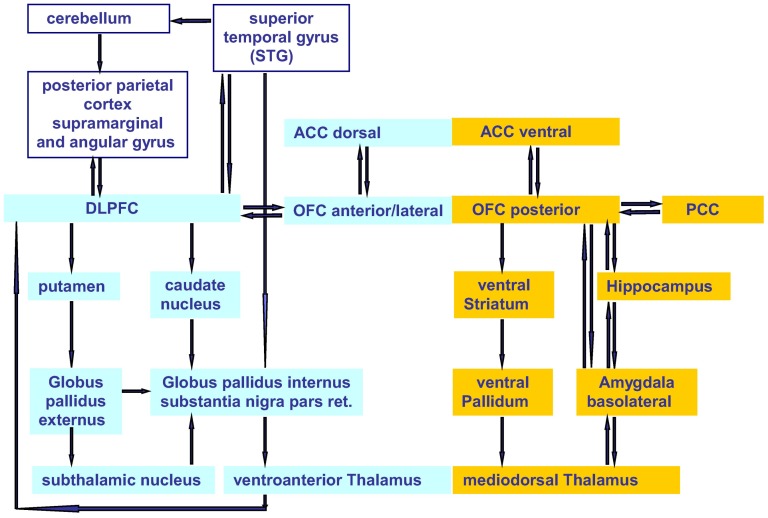
Expanded model of OCD pathophysiology. A primary cognitive network (left) connects prefrontal and ventroanterior thalamic structures via dorsal striatal loops. A primary affective network (right) connects the posterior OFC and ventral parts of the ACC via ventral striatal loops and via limbic structures with the mediodorsal thalamus. The DLPFC interfaces with the parietal cortex and the cerebellum. (Modified according to [14], p. 264; [15], p. 541]).

In more recent models, the classic fronto-striato-thalamic system that proceeds to the dorsal striatum was supplemented by a second network, including the ventral striatum and essential structures of the limbic system [14], [15] (Fig. 1). The central interfaces between these systems are the orbitofrontal cortex (OFC) and the anterior cingulate cortex (ACC). The anterior OFC shows reciprocal connections to the dorsolateral prefrontal cortex (PFC) and the dorsal ACC as well as to the posterior cingulate cortex. The posterior OFC is connected with the ventral part of the ACC, with the amygdale, and the hippocampus. The ventral orbito-striatal network seems to be more active during emotional processes and may be responsible for relaying the emotional OCD components such as fear and anxiety. The dorso-fronto-striatal connections are part of a system which could be responsible for mainly cognitive and executive deficits related to compulsions [13]. Some regions of the parietal cortex (gyrus angularis and gyrus supramarginalis), the cerebellum, and the superior temporal cortex are interconnected via the dorsolateral prefrontal cortex (DLPFC), meaning that there is an interface between the fronto-striatal and the fronto-parietal loop. Because of the resulting activity during symptom provocation and its functions related to attention monitoring and reaction inhibition, the parietal cortex may play a role in controlling obsessive thoughts and compulsive impulses [14], [16].

Therapy options for OCD are the administration of selective serotonin reuptake inhibitors (SSRIs) and psychotherapy. To this end, both behavior therapy with exposure/response prevention and also cognitive behavior therapy have both proven effective in a number of studies [17]–[19]. Several neuroimaging studies have shown the effects of psychotherapy on neuronal activation patterns and brain metabolism [20]–[27]. However, the process of dynamic change itself has not yet been the object of investigation. Those few studies that have been performed by repeatedly using functional MRI in the course of psychotherapy have been conducted in dialectic-behavioral therapy applied to borderline personality disorders [28] and in the psychodynamic therapy of chronic depression [29]. A single-case study on patients with OCD revealed first evidence on discontinuous change of symptom severity preceded by increased dynamic complexity of therapy-related emotions and cognitions, and a pronounced change in neuronal activity during this period [26].

In psychotherapy research there has been a growing interest in the study of patterns of change processes. More frequently, internet-based methods have been used for process monitoring and ambulatory assessment [30], [31]. Meanwhile, converging findings have shown that psychotherapy is a nonlinear process with chaotic dynamics and non-stationarities, i.e., qualitative changes of dynamic patterns [32]–[34], reminiscent of non-equilibrium phase transitions of self-organizing systems [35].

The term “phase transition” originates from physics where it indicates the spontaneous emergence of new qualities or transitions between such qualities in structures or functions of complex systems. Since there are important differences between physical and human systems (such as the kind of driving or control parameters which in human systems are available for experimental manipulation from the outside only in some special cases), we use the term *order transition*. It indicates the qualitative changes of patterns in mental and behavioral systems, which are due to self-organizing processes. In complex non-equilibrium systems, they usually take place in a discontinuous way. This corresponds entirely with practical experience and empirical results on psychotherapeutic change processes, which do not occur in an incremental and linear but in a discontinuous way. Critical moments, the “kairos” (qualified times and occasions for change), specific important experiences or insights, or spontaneous changes of symptom severity have been described repeatedly (for a review of the research on significant events in psychotherapy see [36]). In psychoanalysis, the Boston Change Process Study Group worked on the role of “kairos” and sudden pattern transitions in the tacit procedural knowledge of interpersonal experiences [37]. Most prominent are findings on early rapid responses in psychotherapy [38] which give rise to the renowned research field on sudden changes in psychotherapy [39]–[41].

The study at hand provides evidence of discontinuous transitions in inpatient psychotherapy of OCD patients concerning their subjective experience and the corresponding neuronal activity. Such transitions could be central markers of self-organization processes. Usually, they are introduced by destabilization of current functional patterns and hence by critical dynamic fluctuations, which ought to be recognizable as local time series maxima of dynamic complexity. Therefore, our study combines daily process ratings and their concurrent complexity analyses with repeated high-field functional MRI scans. The main scientific question is whether order transitions within the psychotherapeutic process correspond to changes in neuronal activation patterns.

## Materials and Methods

### Subjects

The study was performed on 9 OCD patients (subtype: washing/contamination fear) (DSM IV: 300.3; ICD10∶7 patients: F42.2 [mixed obsessive thoughts and acts], 2 patients: F42.1 [predominantly compulsive acts]) and 9 healthy controls matched according to age, gender and educational level (Table 1). 8 of the 9 patients had no comorbid psychiatric or somatic diagnoses (clinical judgment based on ICD-10 and DSM-IV criteria and on a psychiatric interview). One patient diagnosed with F42.2 had the additional diagnosis of F34.1 (persistent mood disorder/dysthymia). 8 of the 9 patients were drug-naive. One patient was medicated with an antipsychotic substance (olanzapine, 10 mg/day), an antidepressant (trazodone, 100 mg/day), and a dual serotonergic and noradrenergic reuptake inhibitor (duloxetine, 120 mg/day), with unchanged doses during the study.

**Table 1:** Characterization of patients and healthy controls.

	N	Male/Female	Age	Successful fMRI scans		Days of hospital stay	Diagnoses
			Mean ± *SD*	Number of subjects	Successful fMRI scans	Mean± *SD* (range)	
Patients	9	4/5	31.9±8.3	7	3	55±9.3 (37–65)	7 patients: F42.2
				2	4		(one additionally F34.1)
							2 patients: F42.1
Controls	9	4/5	30.3±8.3	5	3		
				3	2		
				1	4		

Patients and controls did not differ with respect to age, *t*(16) = −0.25, *p* = .802. Length of hospital stay equals the number of daily ratings.

Written informed consent was obtained from all participants after procedures had been fully explained to them, according to the guidelines of the ethics committees of the University of Munich, Germany, or the Medical University of Vienna, Austria.

### Treatment

Patients were treated at two medical centers specialized in the therapy of OCD: The Clinic of Psychosomatic Medicine Windach/Ammersee (Germany) (4 patients) and the Department of Psychiatry and Psychotherapy, Medical University of Vienna (Austria) (5 patients). In both clinics, treatment was performed as inpatient therapy with a planned duration of 8 weeks. Both treatment centers were focused on cognitive behavior therapy with exposure/response prevention (ERP) and were oriented to individual treatment goals. Once per week, two single sessions (each 50 minutes), two group sessions (each 100 minutes), 50 minutes of reflection on the individual Y-BOCS results (Yale-Brown Obsessive Compulsive Scale, [42], [43]), 50 minutes of reflection on the therapy progress, and an individual weekend-planning were offered. The ERP-management took two weeks and was accompanied by a co-therapist. During this period, marital or family sessions were offered. Both therapy concepts (Windach and Vienna) integrated psychoeducation oriented to OCD-related etiological models, but also encouraged the patients to develop individual explanation models of their problems based on their own personal and interpersonal experiences. The treatment rationale of the Vienna University Hospital included also training in social competencies, skills and coping training [44], and a relaxation training. In both centers the treatments were standardized as far as possible, but did not follow a rigid manualization for reasons of final adaptivity and specifity to individual concerns. The therapy concepts are described in detail elsewhere [45], [46].

### Outcome Measures

Therapy results were assessed with the Yale-Brown Obsessive Compulsive Scale (Y-BOCS [42]; German version [43]), the Beck Depression Inventory (BDI [47]), and the Symptom Check-List 90-R (SCL-90-R, German Version [48]) (pre-post comparisons).

### Assessment of Psychotherapy Dynamics

#### Therapy process questionnaire

After admission, patients completed the Therapy Process Questionnaire (TPQ [49], [50]) presented on a PC screen at the end of each day, using the Internet-based Synergetic Navigation System [51]. The TPQ is a self-rating instrument for the assessment of important aspects of therapeutic processes. Daily ratings were given on seven-point Likert scales or visual analogue scales, and then transformed into time series of the clinical course. Explorative factor analysis combined with a validating confirmatory factor analysis of the items resulted in the following subscales of the TPQ: (1) therapy progress, (2) complaints and problem pressure, (3) relationship quality and trust in therapists, (4) dysphoric affect, (5) relationships with fellow patients [50].

#### Dynamic complexity

Dynamic complexity is a newly developed measure to identify non-stationary phenomena and critical instabilities in short time series. Other than variance, complexity describes jumps, volatility and pattern complexity of signals. It is used for the analysis of discrete time series data with a known theoretical data range (for details of the algorithm see [52] and the Appendix S1; for applications on empirical psychotherapy data see [49], [53]). Dynamic complexity combines a fluctuation measure and a distribution measure in a multiplicative way. The fluctuation measure is sensitive to the amplitude and frequency of changes in a time signal, and the distribution measure scans the scattering of values or system states realized within the theoretical range of possible values or system states. In order to identify non-stationarities, the dynamic complexity is calculated within a window of 7 data points ( = 7 days) moving over the time series of each patient. The resulting time series of dynamic complexity was averaged over the items of the TPQ, and the maximum of these dynamics were used as indicators of the most intensive fluctuation periods and the discontinuous transitions during the therapies. Wherever possible, fMRI measurements were performed shortly before or after these transition or symmetry breaking points. It has been shown that order transitions in self-organizing systems are introduced by critical fluctuations and instabilities [35], [49]. For that reason, in the actual study order transitions were identified when the dynamic complexity of daily patient self-assessments (TPQ) was maximal.

### MRI Acquisition

The fMRI measurements were carried out three times (7 patients, 5 controls) or four times (2 patients, 1 control) during the hospital stay or the no-treatment reference period of the controls. 3 controls were scanned only two times. Apart from these exceptions, the healthy controls were scanned with identical time intervals as compared to the matched patients. The process related results of the study refer to the differences between subsequent MRI measurements (inter-scan intervals). The 4 patients recruited from the Clinic of Psychosomatic Medicine Windach and their control subjects were scanned with a 1.5-Tesla MRI scanner (Magnetom Sonata; Siemens, Erlangen, Germany). The 5 patients recruited from the Medical University of Vienna and their controls were scanned with a 3-Tesla MRI scanner (Trio; Siemens, Erlangen, Germany). For all scans a standard head coil was used. A high-resolution T1-weighted scan was acquired for anatomical referencing. Functional images were obtained by a gradient echo-planar imaging sequence (1.5 Tesla MRI: repetition time: 4000 ms; echo time: 53 ms; 16 axial slices; matrix size: 64×64; slice thickness: 6 mm; gap: 0.3 mm. 3 Tesla MRI: repetition time: 2000 ms; echo time: 31 ms; 32 axial slices; matrix size: 64×64; slice thickness: 3 mm; gap: 0.15 mm).

For processing and statistical analysis of the fMRI data, the Brain Voyager Software Package (Brain Innovation, Maastricht, The Netherlands) was used. The first five images were excluded from any further analysis due to relaxation time effects. The preprocessing of the functional data included high-pass filtering (cutoff: three cycles in time course) to low frequency signal drift inherent in echo planar imaging, a slice scan time correction, spatial smoothing (Gaussian filter with FWHM 8.0 mm), and a 3D motion correction. In addition, the functional images were transferred to a standard Talairach brain.

Significant BOLD activity was determined by a cross correlation of MR image pixel intensity with an expected hemodynamic response function. Voxelwise *t*-tests were used to identify those brain areas where the signal change was significantly different between OCD related responses compared to neutral stimuli.

The data of all patients and healthy controls were calculated in the same GLM. For each participant the conditions “OCD-relevant pictures”, “disgust”, and “neutral” were calculated as regressors. In order to quantify local neuronal activity, 8 brain regions were identified to be important for OCD-related neuronal processing. For that purpose, regions of interest (ROIs) were defined which were calculated taking into account the increase of hemodynamic responses during OCD-relevant pictures compared to neutral pictures in patients and controls. ROIs were defined comprising the anterior and medial cingulate cortex and the supplementary motor area (CC/SMA), the dorsolateral prefrontal cortex right (DLPFC r), the dorsolateral prefrontal cortex left (DLPFC l), the insula right (Insula r), the insula left (Insula l), the parietal cortex right (Parietal r), the parietal cortex left (Parietal l), and the cuneus. The number of activated voxels as well as the average activity during the presentation of OCD-relevant information was calculated separately for each participant. Apart from the absolute number of significant voxels per brain area (averaged out over all 9 patients), a weighting was calculated by normalizing the voxels per scan for each patient and the brain area with the largest number of voxels (independent of the scan where this was detected), resulting in brain areas with more voxels receiving more statistical weight (weighted %).

### Picture Material and Stimulation Paradigm

**Figure 2:**
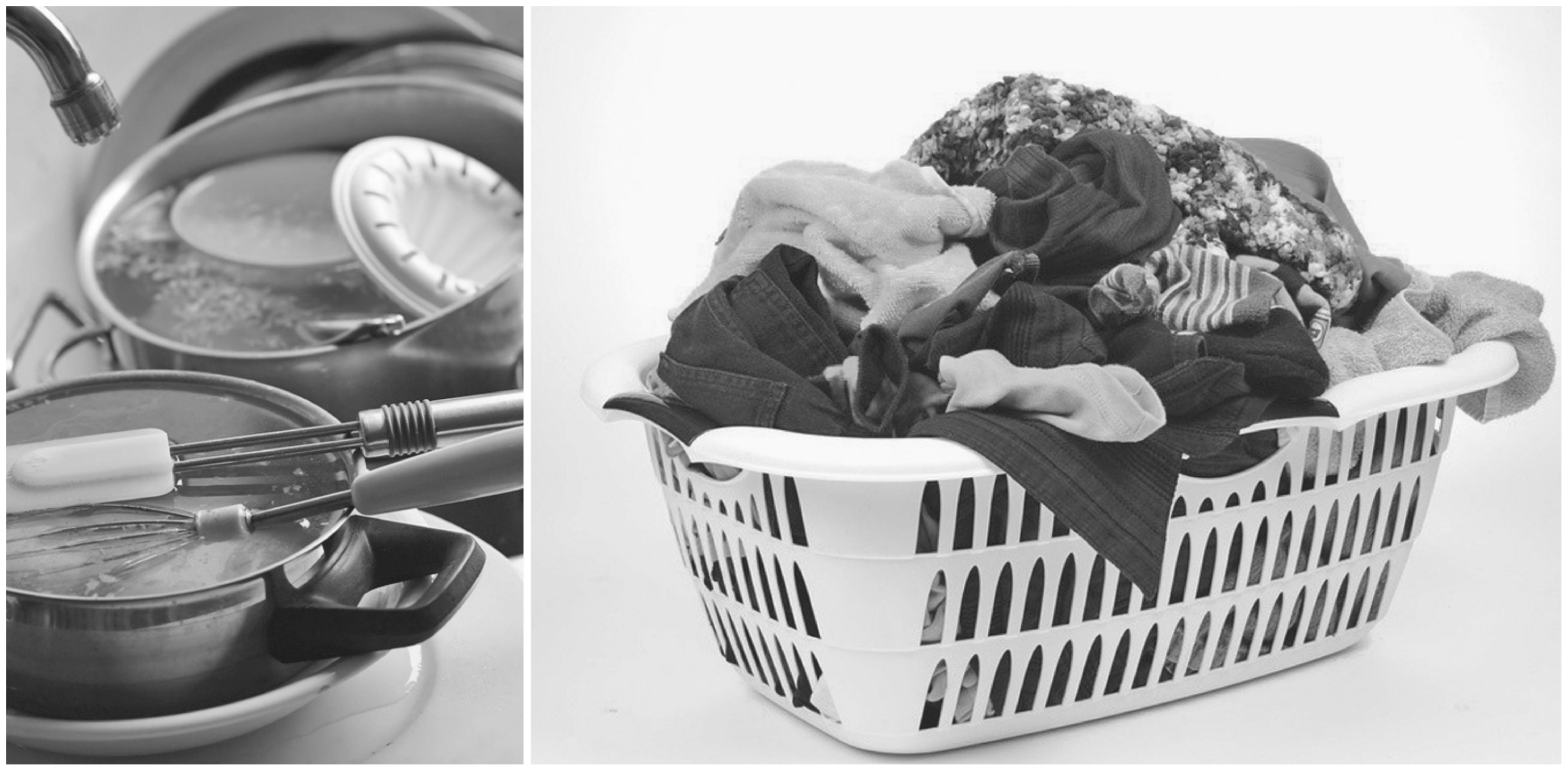
Individualized symptom-provoking pictures. Examples of individual compulsion-triggering pictures from a patients’ home environment. Such pictures are considered trivial for other individuals (for example control subjects). However, to the individual patient with his or her specific background of experience as well as individual conflictual issues, these pictures are emotionally highly charged and symptom provoking.

The visual stimulation consisted of 30 symptom provoking, 30 disgust provoking, and 30 neutral pictures. Disgust and neutral pictures were taken from the International Affective Picture System (IAPS [54]). The OCD-relevant pictures had been photographed by the patients themselves. The pictures showed specific triggers for compulsive behaviors and/or obsessive thoughts (for an example see Fig. 2). The scenes were captured with a digital camera from the patients’ home context (see [55] for first using this individualized symptom provocation paradigm with OCD patients). All pictures were shown twice, four seconds each, in pseudo-randomized order. The same picture sequence was presented to patients and their matched control subjects. All pictures were presented to the participants prior to the first MRI session in order to reduce primacy, surprise, or habituation effects.

The results reported here refer to the difference in brain activation when looking at individualized symptom provoking pictures (ISPP) and standardized neutral pictures (NP) (ISPP> NP). The significance threshold for the 4 patients who were examined with the 1.5-Tesla scanner was set at *p*<.005, for patients who were examined with the 3-Tesla scanner it was set at *p*<.000064, in order to account for the higher sensitivity at 3 Tesla.

Immediately after the MRI sessions, subjects were asked to rate the pictures by means of five-point scales for (a) emotional valence, (b) arousal, and (c) coping/self-efficacy (feeling of being able to cope with the presented situation). The ratings for valence and arousal used the visual symbols of the IAPS standard rating procedure (5 steps). The coping/self-efficacy ratings were also done on a five-point scale (with 1 “not at all” and 5 “very strong”). Table 2 indicates the picture ratings of patients and controls immediately after the first fMRI scan.

**Table 2:** Descriptive results for the picture ratings after the first fMRI.

	Individualized symptom provoking pictures			Disgust pictures			Neutral pictures		
	Valence	Arousal	Coping	Valence	Arousal	Coping	Valence	Arousal	Coping
Patients	1.77±0.28	3.79±0.46	2.41±0.53	1.78±0.34	2.74±1.04	3.96±0.67	3.85±0.66	1.27±0.34	4.98±0.06
Controls	2.94±0.18	1.28±0.34	4.93±0.19	1.68±0.36	3.44±0.82	3.06±1.18	3.49±0.64	1.62±0.54	4.81±0.29
*t*	10.50	−13.23	13.56	−0.61	1.60	−1.98	−1.16	1.69	−1.68
*df*	13.54	14.71	10.04	15.97	15.21	12.66	15.99	13.47	8.59
*p*	<.001	<.001	<.001	.551	.129	.070	.263	.114	.129

Mean and standard deviation (mean ± *SD*) of ratings for the pictures presented (individualized symptom provoking pictures, disgust pictures, neutral pictures) immediately after the first fMRI measurement. Ratings according to valence, arousal, and coping. *t*: *t*-values of two-sided *t*-tests for paired samples.

**Figure 3:**
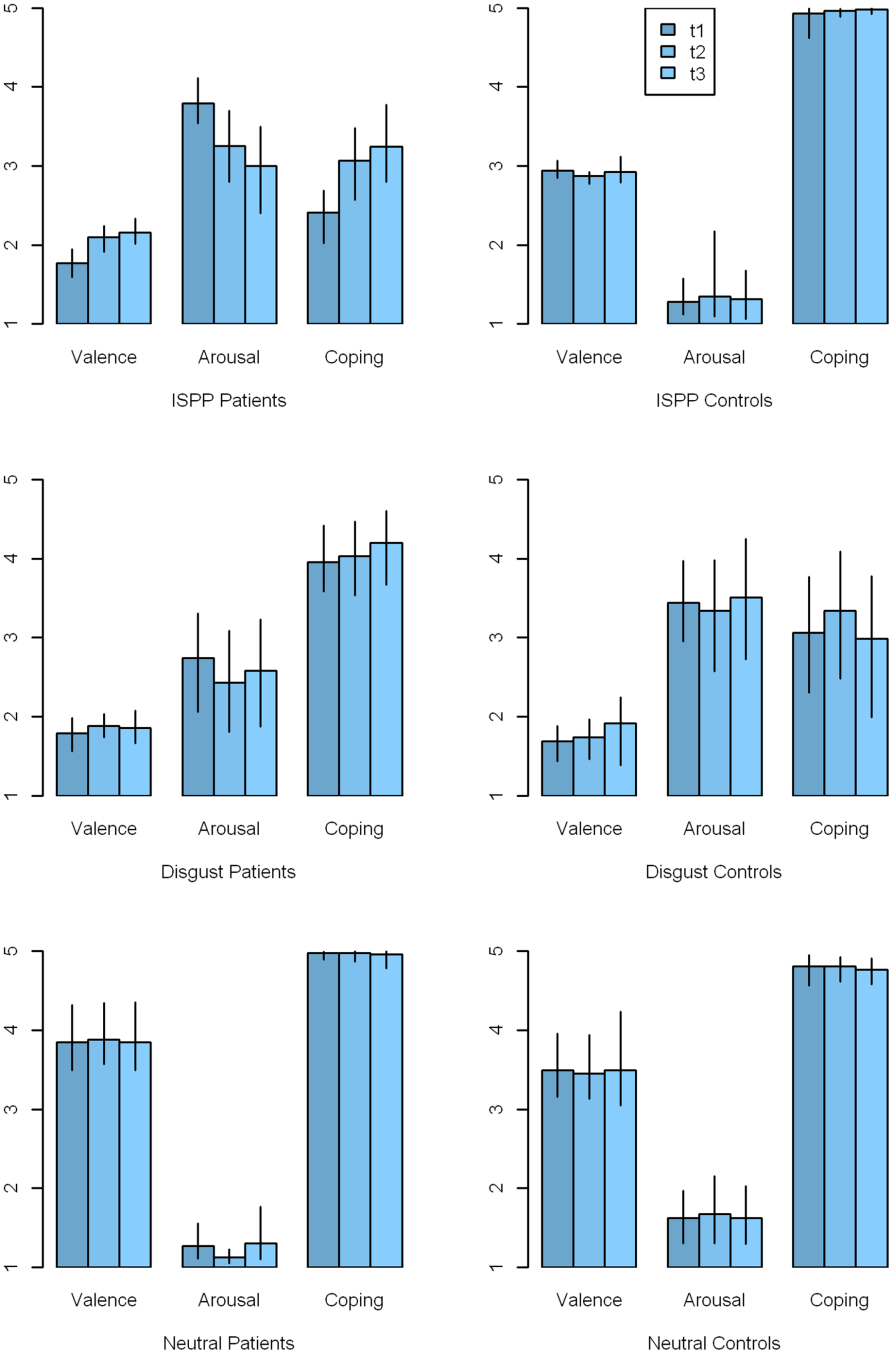
Ratings of stimulus material. Ratings for individualized symptom provoking pictures [a], IAPS disgust pictures [b], and IAPS neutral pictures [c]. Left: patients, right: healthy controls. Rating according to valence, arousal, and coping. The three adjacent columns represent the ratings of three successive fMRI scans. The fourth measurement taken for 2 patients and one control is not represented. 95% confidence intervals of the means were bootstrapped with R’s boot.ci function using 10.000 resamples and the “bca” type of confidence intervals.

Patients rated emotional valence of their individual compulsion-triggering pictures considerably lower than healthy subjects. Arousal was rated considerably higher, and the possibility of handling the situation shown (coping) was rated considerably lower than in healthy controls (all: *p*<.001). Valence ratings did not differ between patients and controls for standardized disgusting pictures. In addition, healthy controls reported a slightly increased arousal (*p* = .129) and less self-efficacy/coping (*p* = .070) than patients during the presentation of disgust-related stimuli. However, the difference was not significant. As far as neutral pictures were concerned, there was no significant difference between patients and controls. In Figure 3, the ratings of all three types of pictures (individualized symptom-provoking, disgust, and neutral) across all fMRI examinations are shown. For patients and controls and for types of pictures, the three adjacent columns represent the ratings of three successive fMRI scans. 95% confidence intervals of the means were bootstrapped with R’s boot.ci function [56], using 10,000 resamples and the “bca” type of confidence intervals. The two scans that were performed with 2 patients at a fourth measurement time were not taken into account in the figure.

Correlations (Pearson’s product-moment correlation *r*) between the three evaluation aspects of the respective symptom provoking pictures were consistent and distinct across all three measurements: Valence with arousal (*r* = −.82, −.83, −.88, all *p*<.001); valence with coping/self-efficacy (*r* = .72,.88,.88, all *p*<.001), arousal with coping/self-efficacy (*r* = −.89, −.87, −.88, all *p*<.001).

### Statistical Analysis

We used paired *t*-tests for the pre-post-comparisons of clinical outcome measures and of brain activity. For the comparisons of brain activity differences within patients (order transitions vs. non order transitions) we used paired *t*-tests, for the comparison of patient’s brain activity differences at order transitions with activity differences from one scan to another (inter-scan differences) of the controls we used unpaired *t*-tests. Additionally, results were validated by non-parametric Wilcoxon tests, which are robust against violations of normal distributions. All these significance-tests were two-sided.

Two-factorial ANOVAs with the factor “group” (patients vs. controls) and the factor “type of transition” (order transition vs. non order transition) were realized. Point-biserial correlations between changes in brain activity and type of transition (order transition vs. non order transition) were calculated to estimate the effect size [57]. Correlations up to.20 are considered small effects, with medium effect sizes ranging from.21 to.35, and large ones are greater than.35.

For all statistics we used the R 2.15.2 [56] except for the ANOVAs, which were calculated with SPSS 13.0. Additional statistical results and the “R” code are available at http://tinyurl.com/c8ebtn6.

## Results

### Therapy Effects

Patients lowered their total Y-BOCS scores from 26.6 at therapy onset to 17.3 at the end of therapy (*p*<.001). BDI results decreased from 22.4 to 12.9 (*p* = .002). The SCL-90-R total score reduction (before: 0.99, after: 0.71) missed the significance threshold (*p* = .079). Assessments of individualized symptom provoking pictures by the patients changed from 1.77 to 2.16 for valence (*p* = .004), from 3.79 to 3.00 for arousal (*p* = .022), and from 2.41 to 3.24 for coping (*p* = .025) (Table 3).

**Table 3:** Pre-post-comparisons of clinical measures, picture ratings, and brain activation.

		Pre	Post	*t* (*df*)	*p*
		Mean ± *SD*	Mean ± *SD*		
Y-BOCS		26.66±8.62	17.33±12.04	6.65 (8)	.000
BDI		22.44±9.32	12.88±7.94	4.62 (7)	.002
SCL-90		0.99±0.49	0.71±0.37	2.05 (7)	.079
ISPP valence		1.77±0.28	2.16±0.26	−3.85 (8)	.004
ISPP arousal		3.79±0.46	3.00±0.89	2.82 (8)	.022
ISPP coping		2.41±0.53	3.24±0.78	−2.75 (8)	.025
CC/SMA	voxel	9949.5±11075.5	201.0±466.1	2.64 (8.02)	.030
	weighted	37.14±34.37	0.89±2.02	3.16 (8.05)	.013
DLPFC r	voxel	1315.9±1425.2	23.4±70.3	2.72 (8.04)	.026
	weighted	4.42±4.32	0.10±0.31	2.99 (8.08)	.017
DLPFC l	voxel	11475.4±8889.6	165.3±293.4	3.82 (8.02)	.005
	weighted	45.88±33.19	0.51±0.80	4.10 (8.01)	.003
Insula r	voxel	3077.8±3916.3	24.2±51.8	2.34 (8.00)	.047
	weighted	10.65±9.60	0.13±0.24	3.29 (8.01)	.011
Insula l	voxel	4619.6±5812.7	14.2±27.0	2.38 (8.00)	.045
	weighted	16.44±17.17	0.07±0.13	2.86 (8.00)	.021
Parietal r	voxel	9991.1±7912.3	985.1±1848.8	3.33 (8.87)	.009
	weighted	40.74±30.02	4.70±8.40	3.47 (9.24)	.007
Parietal l	voxel	15938.7±8503.9	1334.7±1927.1	5.03 (8.82)	.001
	weighted	59.54±25.90	6.62±11.71	5.59 (11.14)	.000
Cuneus	voxel	22628.3±13760.3	3089.7±5547.4	3.95 (10.53)	.002
	weighted	84.21±29.19	16.61±33.22	4.59 (15.74)	.000

Comparison of total scores for Y-BOCS [42], BDI [47], SCL 90-R [48], assessed at the beginning (pre) and at the end of inpatient treatment (post). The ratings for valence, arousal, and coping for individualized symptom provoking pictures, as well as the brain activation (voxel and weighted) were taken at the first (pre) and the third fMRI scan (post). The number of significant voxels (averaged across all 9 patients) of the first and last fMRI measurements were compared separately for eight brain areas (ROIs). Voxel numbers were also relativized for each patient for the brain areas with the largest voxel number, implying that brain areas with larger voxel numbers are weighted more.

Abbreviations: *t*: *t*-values of two-sided *t*-tests. ISPP: individualized symptom provoking pictures; CC/SMA: anterior cingulate cortex/supplementary motor area; DLPFC r: dorsolateral prefrontal cortex right; DLPFC l: dorsolateral prefrontal cortex left; Insula r: insula right; Insula l: insula left; Parietal r: parietal cortex right; Parietal l: parietal cortex left; cuneus.

The neuronal activity within the eight preselected brain regions (i.e., CC/SMA, DLPFC r, l, Insula r, l, parietal cortex r, l, cuneus) differed significantly between the first and the last fMRI measurement. Table 3 shows the absolute number of significant voxels per brain area for the first and the last fMRI measurement, and the weighting of voxels. The weighting was calculated by normalizing the number of voxels per scan for each patient to the brain area with the largest number of voxels (independently of the scan where this was detected). This way, brain areas with more significant voxels received more statistical weight.

The intercorrelation calculated for 9 patients, referring to the activation of the eight brain areas was considerably reduced from therapy onset (mean of all correlations: *r* = .71, *SD* = .09) to the end of therapy (*r* = .29, *SD* = .34). The difference is highly significant: *t* (*df* = 27.0) = 6.10; *p*<.001.

### The Process Perspective: fMRI Correlates of Order Transitions

The identification of order transitions at the maxima of the dynamic complexity of the time series from daily patient self-assessments through the TPQ revealed that 7 patients demonstrated one maximum of dynamic complexity each; these patients participated three times in the fMRI session. 2 patients showed a second maximum in the TPQ rating; these patients were scanned four times. In total, eleven inter-scan-intervals with a therapeutic order transition and nine inter-scan-intervals without such order transitions were observed.

**Figure 4:**
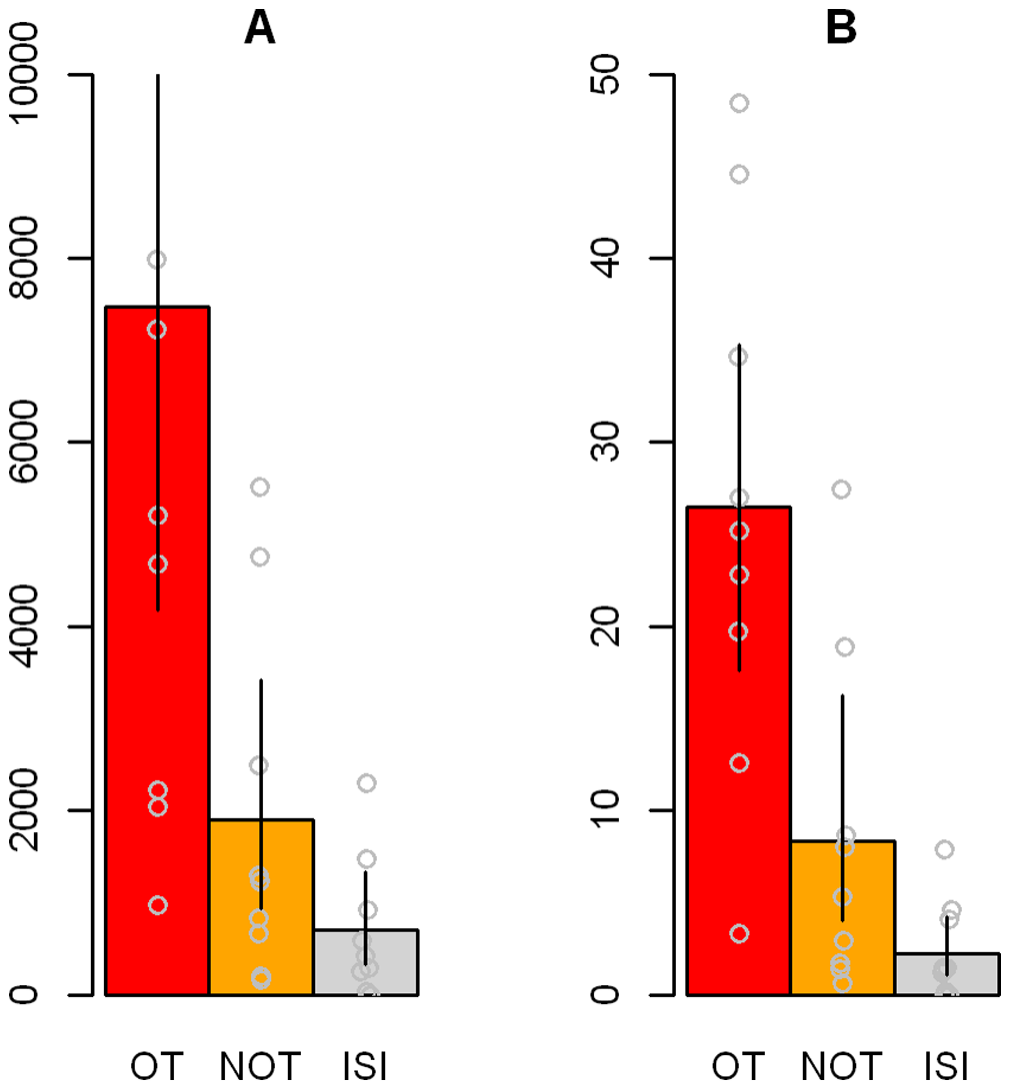
Global changes in brain activity. Differences of brain activity in order transition intervals for patients (red), non order transitions for patients (yellow), and inter-scan-intervals for healthy controls (grey). (a): mean voxel number difference; (b): relative percentual changes. Abbreviations: OT: order transitions; NOT: non order transitions; ISI: inter-scan-intervals for healthy controls.

The changes in numbers of significant voxels for the ISPP> NP contrast revealed greater BOLD response differences in all relevant brain regions during order transition intervals (OT) compared to intervals without order transitions (NOT). As far as the healthy controls are concerned, who underwent neither therapy nor daily self-assessments, the distinction between intervals with and without order transitions is not possible or relevant. Alternatively, in healthy subjects, functional changes were averaged across all inter-scan-intervals (ISI). Figure 4(a) illustrates the changes in significant voxels averaged for the eight brain areas. Figure 4(b) shows the weighted relative change rates in percent, related to the maximum voxel number in one of the brain areas for each patient. This weighted percentage (%) change is supposed to offset the different sensitivity and significance levels caused by measurements with 1.5 and 3 Tesla scanners, respectively. In addition, brain areas with fewer significant voxels are weighted less than those with higher significant voxel numbers. Activation rates and change rates were significantly higher for patients compared to controls.

The differences between types of transition, i.e., between order transition intervals (OT) of patients (mean voxel number difference: 7479, *SD* = 6835) and non order transition intervals (NOT) of patients (mean voxel number difference: 1904, *SD* = 1968, *t* (*df* = 8.00) = 2.13, *p* = .066, Wilcoxon test: *p* = .050) almost reached significance. The number of activated voxels differed significantly between order transition intervals of patients and the inter-scan-intervals (ISI) of the controls (mean voxel number difference: 697, *SD* = 758, *t* (*df* = 8.19) = 2.96, *p* = .017, Wilcoxon test *p*<.001); however, as expected, the difference between the NOT intervals of patients and the ISI intervals of the controls did not differ significantly: *t* (*df* = 10.33) = 1.71, *p* = .116, Wilcoxon test *p* = .161. Similar results were obtained for the relative percentage change: OT (mean difference: 26.48, *SD* = 14.42) vs. NOT (mean difference: 8.34, *SD = *9.13), *t* (*df* = 8.00) = 2.69, *p* = .027; Wilcoxon Test *p* = .027; OT vs. ISI (mean difference: 2.45, *SD* = 2.57), *t* (*df* = 8.45) = 4.98, *p* = .001, Wilcoxon Test *p*<.007); NOT vs. ISI: *t* (*df* = 9.12) = 1.95, *p* = .083, Wilcoxon Test: *p* = .040.

**Figure 5:**
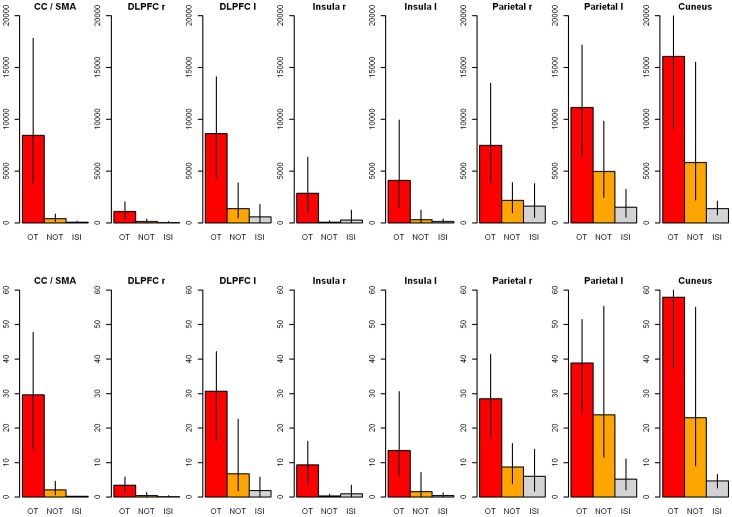
Changes in brain activity in eight regions of interest. Differences of brain activity in order transition intervals for patients (red), non order transitions for patients (yellow) and inter-scan-intervals for healthy controls (grey). Top: mean voxel number difference; below: relative percentage changes. Abbreviations: OT: order transitions; NOT: non order transitions; ISI: inter-scan-intervals for healthy controls. CC/SMA: anterior cingulate cortex/supplementary motor area; DLPFC r: dorsolateral prefrontal cortex right; DLPFC l: dorsolateral prefrontal cortex left; Insula r: insula right; Insula l: insula left; Parietal r: parietal cortex right; Parietal l: parietal cortex left; cuneus.

Table 4 and Figure 5 illustrate neuronal activation for order transition intervals in patients (OT), non order transition intervals in patients (NOT) and inter-scan-intervals in controls (ISI) for all eight brain areas. Pronounced differences were demonstrated between OT and NOT, particularly for weighted percentage changes (w % change), and even more clearly for OT vs. ISI, but not for NOT vs. ISI. The most pronounced differences are noticeable for the CC/SMA, the DLPFC left, DLPFC right and insula right. For these regions there were also significant interaction effects in related ANOVAs (“group” [patients vs. controls]×“type of transition”[OT vs. NOT]) (see Table 4 and additional results online). The equally pronounced differences in the area of the cuneus and the left parietal cortex did not reach significance due to the NOTs’ wide confidence intervals. The high individual variability is a result from distinctly differing change patterns in patients as well as therapy processes. However, most patients (i.e., >75%) showed clearly recognizable order transitions in different brain areas.

**Table 4:** Differences in brain activation in relation to order transitions.

		OT-NOT				OT-ISI				NOT-ISI			
		Order transitions in patients (OT) vs.				Order transitions in patients (OT) vs.				Non order transitions in patients (NOT) vs.			
		non order transitions (NOT) in patients				inter-scan-intervals in controls (ISI)				inter-scan-intervals in controls (ISI)			
		mean difference	*t* (*df* = 8)	*p*	*r*	mean difference	*t* (*df*)	*p*	*r*	mean difference	*t* (*df*)	*p*	*r*
Mean of all	diff voxels	5575.10	2.13	.066	.51	6782.13	2.96 (8.19)	.017	.59	1207.03	1.71 (10.33)	.116	.39
brain areas	w % change^a^	18.14	2.69	.027	.62	25.05	4.98 (8.45)	.001	.79	5.89	1.95 (9.12)	.083	.44
CC/SMA	diff voxels	8045.83	2.34	.047	.50	8387.56	2.43 (8.00)	.041	.52	341.72	1.74 (8.54)	.119	.40
	w % change^a^	27.53	3.14	.014	.60	29.38	3.20 (8.00)	.013	.63	1.86	1.84 (8.14)	.102	.43
DLPFC r	diff voxels	977.06	2.29	.051	.48	1054.39	2.42 (8.05)	.042	.52	77.33	0.92 (9.57)	.380	.22
	w % change^a^	3.01	2.68	.028	.53	3.30	2.80 (8.14)	.022	.59	0.29	0.95 (10.36)	.362	.25
DLPFC l	diff voxels	7228.61	2.57	.033	.54	8025.56	2.97 (8.37)	.017	.60	796.94	0.93 (12.19)	.372	.23
	w % change^a^	23.90	2.95	.018	.59	28.73	4.97 (8.54)	.003	.71	4.83	1.06 (9.34)	.320	.26
Insula r	diff voxels	2792.61	2.07	.072	.46	2590.41	1.90 (8.51)	.091	.43	−202.20	−0.83 (8.43)	.429	.20
	w % change^a^	9.03	2.67	.028	.56	8.43	2.45 (8.97)	.037	.52	−0.60	−0.71 (8.73)	.496	.18
Insula l	diff voxels	3811.72	1.83	.103	.42	3966.89	1.98 (8.03)	.083	.44	155.17	0.62 (9.71)	.552	.15
	w % change	11.85	1.85	.101	.44	12.97	2.19 (8.03)	.060	.48	1.13	0.80 (8.60)	.445	.20
Parietal r	diff voxels	5328.22	1.98	.083	.45	5862.83	2.23 (9.70)	.050	.49	534.61	0.47 (15.97)	.643	.11
	w % change	19.83	2.34	.047	.56	22.49	3.10 (11.18)	.010	.61	2.66	0.62 (15.99)	.545	.15
Parietal l	diff voxels	6164.06	1.63	.142	.41	9643.78	3.28 (8.87)	.001	.63	3479.72	1.78 (10.09)	.105	.40
	w % change	14.98	1.17	.274	.29	33.61	4.33 (9.42)	.002	.73	18.63	1.79 (8.76)	.108	.41
Cuneus	diff voxels	10252.67	1.53	.163	.41	14725.61	3.03 (8.09)	.016	.60	4472.94	1.45 (8.22)	.185	.34
	w % change	35.00	1.87	.098	.52	53.26	5.33 (8.18)	.001	.80	18.26	1.71 (8.16)	.125	.40

Differences between BOLD responses before and after order transitions for patients [OT], before and after non order transitions for patients [NOT], and differences between fMRI scans for healthy controls (ISI: inter-scan-intervals). Differences between these comparisons are shown for OT vs. NOT, OT vs. ISI, and NOT vs. ISI.

Abbreviations: diff. voxels: Differences in voxel changes. w% change: weighted percentage change, *t*: *t*-values of two-sided *t*-tests, *r*: point -biserial correlations which are added as measure of effect size. Correlations up to.20 are considered small effects, with medium effect sizes ranging from.21 to.35, and large ones are greater than.35.

a For these regions the interaction effects between the factors “group” (patients vs. controls)×“type of transition” (OT vs. NOT/ISI) in related ANOVAs were statistically significant (results are located here: http://tinyurl.com/c8ebtn6).

The point -biserial correlation between changes in voxel numbers (per patient, and averaged out over the eight brain areas) during order transitions (OT) and outside of order transitions (NOT) is.51 (*p* = .066); the point -biserial correlation between the relative percentage changes weighted for voxels (w % change) of the brain regions is.62 (*p* = .027). These results indicate large effect sizes [57]. Table 4 illustrates the point -biserial correlations beyond OT-NOT comparisons for other comparisons (i.e., OT-ISI, NOT-ISI), but also the comparisons of separate point -biserial correlations for all eight respective brain areas.

**Figure 6:**
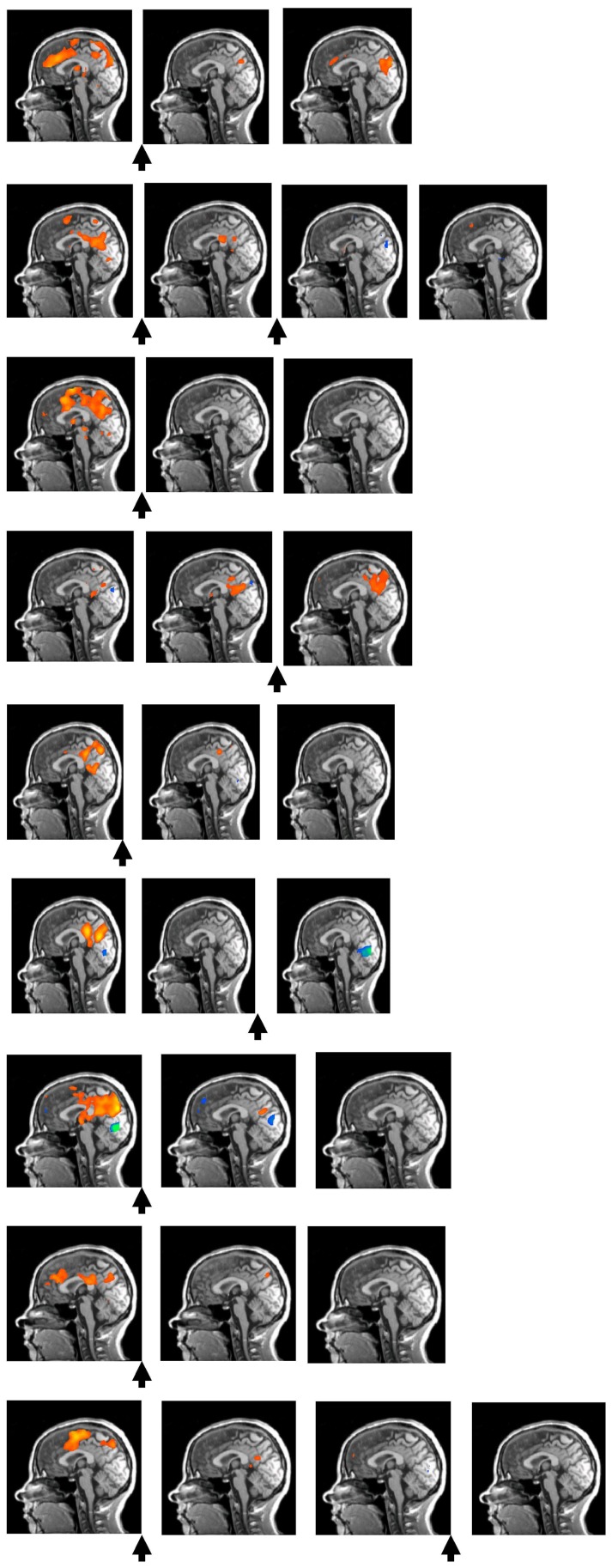
Activation patterns for each patient during fMRI scans in sagittal views. Talairach coordinate: *x* = 0. The order transitions within individual therapies are marked by arrows.

Figure 6 shows the activation patterns for all 9 patients during individual fMRI scans (Talairach coordinate: *x* = 0). Order transitions for individual patients are marked by arrows.

Not only the activation of individual brain regions, but also their intercorrelations show temporal dynamics. The high average intercorrelation of the eight brain regions during the first fMRI measurement (*r* = .71, *SD = *.09) is already lower during the second scan (*r* = .39, *SD* = .32, *p* of the difference <.001); during the third scan it became even lower (*r* = .29, *SD* = .34, *p* of the difference = .310). This corresponds to the finding that in 7 out of 9 patients, order transitions became apparent between the first and the second fMRI measurement. When comparing correlations before and after order transitions, the difference is even more striking (independent of where the order transitions are located in the course of therapy): It changed from *r* = .73 (*SD* = .09) to *r* = .33 (*SD* = .33) (*p* of the difference <.001). In addition to the reduced correlation, a differentiation of intercorrelations occurred which is reflected in an increase in variation, i.e., the standard deviation of the intercorrelations increased from *SD* =  = .09 to *SD* = .33.

## Discussion

This study demonstrates that psychotherapy efficiently works in treating obsessive-compulsive disorders. The changes mainly affect the anterior and medial cingulate cortex/supplementary motor area (CC/SMA), the left and right dorsolateral prefrontal cortex (DLPFC l, r), and the right insular cortex (Insula r). Our findings corresponds with those of other studies [20]–[27], which essentially showed that both, pathological hyper- or hypofunction of neuronal networks involved in compulsion-specific behaviors normalize in the course of therapy.

Actual psychological change processes were documented via daily self-assessment with the Therapy Process Questionnaire. The self-rating data were related to changes in neuronal activation patterns and demonstrate that the most concise changes in brain activity occur in temporal proximity of order transitions. In synergetics and complexity science, order or phase transitions indicate the spontaneous emergence of new collective patterns or qualitative shifts of such patterns in complex, nonlinear systems [35]. Although we identified only one order transition in most of the cases (i.e., in 7 out of 9), we do not suggest that psychotherapy consists in a simple transition from a pathological state to a physiological or healthy state. It can be assumed that there are several stable regimes characterizing healthy functioning and even more than one pathological state. Psychotherapy does not only trigger movements of patient’s behavior in a multi-attractor landscape but gives rise to emerging new attractors in a potential landscape of increasing complexity. A higher scan frequency during the change process could provide more detailed insight into cascades of such order transitions.

Changes in the activity of brain areas outside of order transitions are considerably lower than changes during order transitions, similar to the differences between fMRI scans from healthy controls, who did not undergo psychotherapy and did not experience any dynamic order transitions. Results support the assumption that psychotherapeutic processes occur in the form of discontinuous changes, as postulated by the theory of complex, self-organizing systems [35], [49], [58]–[60]. According to this model, psychotherapy is the procedural creation of conditions enabling biopsychosocial self-organization processes. The strong relationship between order transitions and BOLD responses observed in the present study reversely proves the operationalization of order transitions through the maximum of dynamic complexities of the time series, as gained from daily self-assessment by using the Synergetic Navigation System.

From 20 inter-scan-intervals during psychotherapy, 11 were identified as order transitions, 7 of which occurred between the first and the second fMRI measurement. This corresponds to findings of so-called early rapid responses, which demonstrate that changes in symptom and problem intensity occur discontinuously and mainly during early phases of the psychotherapeutic process. Surprisingly, these changes frequently occur *before* main interventions are applied, for example, cognitive restructuring before the implementation of specific methods from cognitive behavior therapy [38], [39]. Stiles et al. [40] found sudden improvements among clients with a variety of disorders treated by a variety of approaches in routine clinical settings; Stulz et al. [41] report on early changes in routine outpatient conditions. As demonstrated by a single case report on the psychotherapy process of an OCD patient [26], the steepest gradient of symptom reduction and a qualitatively different brain activity pattern occurred before main interventions (such as exposure with response prevention ERP), were implemented. Sudden changes seem to be a robust and universal phenomenon in psychotherapy. A substantial percentage of patients experience discontinuously shaped patterns of change (e.g., [49], [60]).

In future studies it should be investigated in more detail whether order transitions in early phases of the change process are a substantial characteristic of psychotherapies or not. One alternative explanation could be neural habituation effects as described in social anxiety disorders (concerning limbic structures and OFC, see [61]).

It has been shown that stimulation through individualized pictures for the purpose of symptom provocation results in effects distinctly different from neutral, but also from standardized disgust-eliciting pictures. There are other studies that have investigated the results of individualized, system-provoking material, such as pictures from patients’ personal environment [55], verbal descriptions of compulsion-related objects [23], or tactile stimulation through “dirty” objects [62], [63]. Buchheim et al. [29] used individualized scripts of attachment-relevant themes, which were used to provoke attachment-relevant conflict patterns in repeated fMRI scans during the psychoanalytic process of treating chronically depressed patients. By investigating the role of cortico-amygdala circuits in the pathophysiology of OCD, Simon et al. [11] collected a great variety of symptom triggers (which were thematically related to aggressive obsessions, contamination/washing, checking, symmetry/ordering, hoarding, and counting), where patients, in addition to exposure to standardized aversive and neutral pictures, were supposed to provide responses according to the degree of induced obsessive states, anxiety, valence and arousal. Meanwhile, the Maudsley Obsessive-Compulsive Stimuli Set provides a specific and standardized paradigm for symptom-specific provocation of OCD states [64], which in future could enable the application of individual and standardized pictures to be used in a combined paradigm.

Several of the ROIs investigated in this study are part of a comprehensive OCD-related neuronal network [6], [13]–[15], [65], [66] and have been identified as change-relevant in a number of outcome studies on the psychotherapy of OCD (e.g. [22], [23], [25]). For the purpose of quantifying changes, rather large ROIs were selected, which made it impossible to focus on smaller basal ganglia structures (e.g., the head of the nucleus caudatus [20], [24]), or areas of the amygdala [11], [67]. Other brain regions that have been related to OCD symptoms before did not show reliable responses in the present study, for example the ventromedial and the orbitofrontal cortex.

### Limitations

This study has a number of methodological limitations. Like numerous other fMRI-based psychotherapy studies, the sample size (9 patients, 9 controls) is small. This is partly due to restrictive inclusion criteria limited to drug-naive patients of the washing/contamination fear subgroup of OCD with no additional Axis I disorder. Compiling a sample according to these criteria is difficult. Patients had to be willing to undertake daily self-ratings and repeated fMRI scanning. Despite of this restricted sample size, the results of the study are not vague, but statistically significant and straightforward. Another limitation is that neither psychological nor fMRI-based follow-up exams were possible. Other problems may result from the fact that some of the patients were scanned in a 1.5 Tesla scanner and some in a 3 Tesla scanner, also using different head coils. This created a bias in measurement sensitivity, which was counterbalanced by calculations of weighted percentage changes as described above. However, the lower sensitivity of the 1.5 Tesla scanner did not dilute the results.

The dimension of poor versus good insight could interfere with the results since there seem to exist neuronal correlates of different insight levels [65], [68]. Although patients were classified to the good insight level by their psychiatrists (all patients were able to take part in a cognitive behavioral treatment program) we did not apply a systematic measure of the poor vs. good insight dimension, since studies showed that the degree of insight did not predict the likelihood of response to psychotherapy [69]. This and other possible therapy modulating covariates (as trait and state anxiety) will be included in a replication study, which offers the possibility to take into consideration the shortcomings of this study.

### Outlook

The replication of the study will include a larger sample size and another drug-free diagnostic group (such as major depression) in addition to a healthy control group in order to distinguish between OCD-specific activation patterns on the one hand, and diagnosis-independent neuronal mechanisms during therapeutic change processes (e.g., order transitions) on the other hand. Furthermore, it would be interesting to perform the scans precisely during periods of low and high dynamic complexity and then compare order transitions with non order transitions as well as phases of critical instability with phases of relative stability. This comparison would be of great theoretical and clinical importance because critical instabilities represent the sensitive phases of therapies. The question would be whether neuronal correlates of instability during therapeutic change processes could be identified. This kind of investigation could focus on the cingulate cortex, which plays an important role as conflict monitoring system (apart from other functions like somatosensoric integration, mediation of affective and cognitive processes, control of attention, or processing of painful stimuli). Especially the dorsal part of the ACC is sensitive to ambiguous or conflicting information [70], [71], is involved in decision making [72], [73], and its activation is predictive to treatment outcome in depression [74]. Therefore, its activity may be an indicator of symmetry states in brain functioning, which are characterized by two or more dynamic patterns or attractors in competition. Using a movement coordination paradigm modeled by Haken, Kelso, and Bunz [75], Meyer-Lindenberg and co-workers [76] were able to demonstrate neuronal correlates of instability and symmetry breaking processes in the motoric brain. Evidently, non-equilibrium systems like the brain are governed by (in-)stability in response to only small disturbances (in the Meyer-Lindenberg study realized by varying degrees of transcranial magnetic stimulation). More generally, we would expect to find instability correlates not only in specific brain areas but in the functioning of networks. This could be crucial in understanding psychotherapy, since change processes seem to be driven by rather small interventions during instability states.

At any rate, it would make sense to track the changes related to effective connectivity of OCD-specific neuronal networks in psychotherapeutic processes, which could be achieved by Dynamic Causal Modeling (DCM, [77]–[79]) or related methods. Using DCM, an error and conflict monitoring system for OCD could be specified, describing reciprocal connectivity between the left DLPFC, right DLPFC, rostral and dorsal ACC [80]. Basic analyses of structural, functional, and effective connectivity used in systems neuroscience [81], together with results gained from increased theta band activities in OCD – as seen in the medial-ventral PFC, the temporal pole and the parieto-occipital cortex [82] – should provide new therapeutic insights. In case such insights indicate that a pathological hypersynchronization and connectivity in OCD-relevant neuronal networks ([15], see Fig. 1) does indeed exist, non-invasive sensory desynchronization stimulation as used in Coordinated Reset therapy [83] (successfully used for treating chronic tinnitus [84]) might provide promising results also for OCD.

The effect of non-invasive CR stimulation is a resynchronization of pathologically oversynchronized populations of neurons. CR counteracts abnormal neuronal interactions detuning the macroscopic frequency of the collective oscillators – which is the abnormally established order parameters of neural synchronization – and by doing this, it restores the naturally varying frequencies of the individual oscillatory units. Neurons restore the range of physiological functioning and can engage in changing and varying synchronization patterns. Consequently, the coupling strengths of the synapses (synaptic weights) are reduced and a long term rewiring of neuronal networks is reached. Non-invasive neuromodulation could take the role of unlearning pathological network activity in OCD, whereas psychotherapy could take the role of new learning of changed network patterns preparing changed cognitive, affective, and behavioral functioning.

For the future of psychotherapy research it seems promising that the theory of self-organization in complex systems has proven to be not only a theory of pattern formation in physics, but a general theory of structures and a conceptualizing module for modeling and thinking in quite different disciplines. Its general concepts, equations, and mathematical formalisms successfully founded a transdisciplinary framework of modern science. In psychotherapy it could have the potential for an integration of different converging streams: (i) systems neuroscience, which investigates nonlinear network dynamics of the brain, (ii) developments in internet-based therapy monitoring and therapy feedback, (iii) process-outcome-research focusing on sudden gains, crisis-repair dynamics, and other nonlinear phenomena, (iv) the contextual model of psychotherapy focusing on common factors instead of treatment techniques, and (v) actual trends in psychodynamic therapy, which accentuate critical moments of interpersonal experiences transforming the procedural knowledge of patients on attachment patterns. These converging developments actually are forming the “Gestalt” of an integrative, dynamic neuro-psychotherapy.

## Supporting Information

Appendix S1 **Algorithm of dynamic complexity.**
(DOCX)
