# Supplementary material for: Discontinuous Patterns of Brain Activation in the Psychotherapy Process of Obsessive-Compulsive Disorder: Converging Results from Repeated fMRI and Daily Self-Reports
Source: PLoS One. 2013 Aug 15;8(8):e71863. doi: 10.1371/journal.pone.0071863 (PMC3744482; doi:10.1371/journal.pone.0071863)
Supplement: Appendix S1 — Algorithm of dynamic complexity. (DOCX) [file pone.0071863.s001.docx]

**APPENDIX**

An algorithm of dynamic complexity was used in this article to identify non-stationary phenomena and critical instabilities in short and coarse-grained time series. Dynamic complexity is the multiplicative product of a fluctuation measure and a distribution measure. Both measures are used for the analysis of discrete time series data with known theoretical data ranges. Let *x_t_* represent the value of a variable measured at time *t* on the basis of a constant and discrete time interval (scanning frequency, e.g., one observation per day). Values are represented on the basis of a constant unit with theoretical data range *s* between the theoretical minimum *x_min_* and the theoretical maximum *x_max_* of *x*. The fluctuation measure (*F*) is sensitive to the amplitude and frequency of changes in a time signal, and the distribution measure (*D*) scans the scattering of values or system states realised within the theoretical data range of possible values or system states. In order to identify non-stationarity, the two measures are calculated within a data-window moving over the time series.

Fluctuation Intensity

The fluctuation algorithm is applied to segments of discrete time series. These segments are defined by the width of a moving window that can be fixed arbitrarily. The window runs over the whole time series and results in a continuous fluctuation intensity measure *F*. All measurement points within the window are subdivided into periods with cut-off points defined by changes in slope (points of return *k*). Trends can be: “increasing”, “decreasing”, or “no change” (see Figure 7 below). The difference between the values *x*_n_ at the points of return *k* is taken irrespective of the sign – in absolute terms: – and is divided by the duration of the period (i.e., the number of data points within the period from one point of return *k* to the next one *k*+1). By this, the change rate is related to its duration, and *F* is sensitive to the frequency as well as to the amplitude of the fluctuation. Theses fractions are summed up within the window. In order to normalise the fluctuation intensity, the result is related to the greatest possible fluctuation which is given by the maximum amount of change within a minimum duration. This is the sum of the differences between the lowest and the highest value of the available range between one and the next measurement point. The formula results in normalised fluctuation intensity 0 ≤ *F* ≤ 1:

(1) $F=\frac{\sum_{i=1}^{I} \frac{y_{i}}{(n_{k+1}-n_{k})}}{s(m-1)}$

with

$$y_{i}=\left|x_{n_{k+1}}-x_{n_{k}}\right|$$

*x_n_* *n*^th^ value of the time series

*k* points of return (number of changes in slope of the data sequence)

*i* periods between points of return

*I* total number of such periods within the window

*m* number of measurement points within a moving window

*m*–1 number of intervals between all measurement points of a window

*s = x_max_ - x_min_* with *x_min_* smallest value of the scale, *x_max_* largest value of the scale.

One can immediately derive that $\sum_{i=1}^{I} \frac{y_{i}}{n_{k+1}-n_{k}}\leq s(m-1)$, so 0 ≤ *F* ≤ 1, see Figure 7.

Note that *s*(*m*-1) is the window area expressed in units of *t* and *x* and that *s* is not the empirical data range but the theoretical range of the scale.

Figure 7: **Illustration of the fluctuation algorithm.** In this example, the *F*-value is calculated as follows: The first contributor is between *k*=1 and *k*=2 a difference of 3 (since *x*_1_ = 3, *x*_3_ = 6), divided by 2 (2 is the number of intervals between *k*=1 and *k*=2). The next contributor is between *k*=2 and *k*=3 a difference of 3, divided by only one interval. Next is a difference of 0 divided by 2 intervals (remains 0) and the last difference between *k*=4 and *k*=5 is 4, divided by 1. So we sum up 3/2 + 3/1 + 0/2 + 4/1 = 8.5. This sum is divided by the maximum of possible fluctuation, which in this case is (with the greatest number of points of return) *k* = 1 to *k* = 7, and *s* = (*x_max_* – *x_min_*) = 7 – 1 = 6): 6/1 + 6/1 + 6/1+ 6/1 + 6/1 + 6/1 = 36. F = 8.5/36 = .23611.

Distribution

The degree of distribution *D* represents another aspect of critical instabilities. Whereas *F* is at its maximum when the dynamics jump between the minimum and maximum values with greatest and equal frequency, instabilities are often characterised by irregularities, resulting in quite different system states. In the extreme case, the values should be irregularly and chaotically distributed across the range of the measurement scale. As a result, the degree of distribution measures the deviance of the values from an ideal equal distribution of the values across the range or measurement scale. As for the calculation of *F*, we used a moving window running through the whole process and by doing this we consider the values over the full course of the process. For the distribution measure the order of values within the moving window is irrelevant, and in a first step values are sorted in ascending order. Let *x_i_* be the values of a variable *x* at the sorting position *i* within the ≤moving window *X* which is given by:

X = {x_1_, x_2_, x_3_, ... x_m_} with x_1_ ≤ x_2_ ≤ x_3_ ≤ ... x_m_

In the following calculation this sorted window is compared with an artificial data set of equally distributed values. This artificial data set consists of the same number *m* of values arranged in ascending order in equally spaced intervals between the theoretical scale minimum and maximum. The interval *I* is given by *I*=*s* / (*m*-1), *s = x_max_ - x_min_* and the artificial data set *Y* is given by:

*Y* = {*y*_1_=*I**1, *y*_2_=*I**2, *y*_3_=*I**3, ... *y_m_*=*I***m*}

If the data set in *X* is equally distributed within the data range, then differences between values at different positions in *X* must be equal to the differences in *Y* at the same positions. To give an example, if *X* is perfectly equally distributed within the data range, then δ*_Y_*_.2-1_ = *y*_2_-*y*_1_ = δ*_X_*_.2-1_ = *x*_2_-*x*_1_. Generally the aberration Δ*_ba_* of *X* from the ideal given in *Y* can be calculated for the positions *a* and *b* as follows:

Δ*_ba_*= δ*_Y.b-a_* – δ*_X.b-a_* with δ*_Y.b-a_* = *y_b_-y_a_* and δ*_X.b-a_* = *x_b_-x_a_*

In total the aberration Δ* is given by the following permutation of a and b.

Δ* = $\sum_{c=1}^{m-1} \sum_{d=c+1}^{m} \sum_{a=c}^{d-1} \sum_{b=a+1}^{d} \Delta_{ba}\Theta(\Delta_{ba})$

The two outer sums are permutations of all combinations of *c* and *d* within the window. The inner sums of *a* and *b* are representing all combinations of positions within the interval given by *c* and *d*.

Θ (Δ*_ba_*) is the Heaviside step function resulting in 1 if Δ*_ba_* is a positive number; otherwise the function results in 0. Therefore, only positive aberrations are considered, because negative aberrations have the consequence of resulting in positive ones in other positions.

Hence, the distribution measurement *D* is given by:

(2) *D* = 1 – $\sum_{c=1}^{m-1} \sum_{d=c+1}^{m} \sum_{a=c}^{d-1} \sum_{b=a+1}^{d} \frac{\Delta_{ba}\Theta(\Delta_{ba})}{\delta_{Y.ba}}$

One can see that *D* is normalized so that 0 ≤ *D* ≤ 1, and a high value of *D* are the result of equally distributed measures of *x* within the moving window.
